# acmgscaler: an R package and Colab for standardized gene-level variant effect score calibration within the ACMG/AMP framework

**DOI:** 10.1093/bioinformatics/btaf503

**Published:** 2025-09-10

**Authors:** Mihaly Badonyi, Joseph A Marsh

**Affiliations:** MRC Human Genetics Unit, Institute of Genetics and Cancer, University of Edinburgh, Edinburgh EH4 2XU, United Kingdom; MRC Human Genetics Unit, Institute of Genetics and Cancer, University of Edinburgh, Edinburgh EH4 2XU, United Kingdom

## Abstract

**Motivation:**

A genome-wide variant effect calibration method was recently developed under the guidelines of the American College of Medical Genetics and Genomics and the Association for Molecular Pathology (ACMG/AMP), following ClinGen recommendations for variant classification. While genome-wide approaches offer clinical utility, emerging evidence highlights the need for gene- and context-specific calibration to improve accuracy. Building on previous work, we have developed an algorithm tailored to converting functional scores from both multiplexed assays of variant effects (MAVEs) and computational variant effect predictors (VEPs) into ACMG/AMP evidence strengths.

**Results:**

Our method is designed to deliver consistent performance across different genes and score distributions, with all variables adaptively determined from the input data, preventing selective adjustments or overfitting that could inflate evidence strengths beyond empirical support. To facilitate adoption, we introduce acmgscaler, a lightweight R package and a plug-and-play Google Colab notebook for the calibration of custom datasets. This algorithmic framework bridges the gap between MAVEs/VEPs and clinically actionable variant classification.

**Availability and implementation:**

The R package and Colab notebook are available at https://github.com/badonyi/acmgscaler.

## 1 Introduction

To establish a unified approach to clinical genetic and genomic testing, the American College of Medical Genetics and Genomics and the Association for Molecular Pathology (ACMG/AMP) outlined guidelines for assessing the pathogenicity and benignity of variants in Mendelian disease genes (Richards *et al.* 2015). These guidelines have since been formalized within a probabilistic framework, demonstrating how ACMG/AMP criteria for combining clinical evidence align with likelihood ratios for pathogenicity (Tavtigian *et al.* 2018). Within this framework, the strength of evidence for each classification (supporting, moderate, strong, and very strong) has been defined to increase exponentially. This model has provided the foundation for systematically validating and calibrating pathogenicity evidence, advancing efforts to refine variant classification and extend the role of predictive tools beyond supporting-level evidence (Brnich *et al.* 2019).

One such extension applies to computational variant effect predictors (VEPs), where appropriate weighting of evidence is integral to ensuring accurate classification. Recently, Pejaver *et al.* developed a method to estimate the pathogenic-over-benign density ratio for a variant by applying a narrow sliding window to a sorted list of scores labelled as pathogenic or benign (Pejaver *et al.* 2022). The resulting odds of pathogenicity, equivalent to positive likelihood ratios (LR+; hereafter referred to as LR), can be compared to evidence thresholds established by the ACMG/AMP framework under a predefined prior probability of pathogenicity (Richards *et al.* 2015, Tavtigian *et al.* 2018, Brnich *et al.* 2019). Using this approach, the authors estimated genome-wide thresholds, i.e. score intervals, for each evidence level across 13 widely used VEPs. This work has since been extended with the calibration of additional VEPs (Bergquist *et al.* 2025).

Applying genome-wide calibration to VEP scores for variant classification has demonstrated notable benefits. For example, one study used genome-wide thresholds in 300 probands with suspected rare disease and found a 2.6-fold increase in variants reaching strong evidence for pathogenicity compared to when only established disease-associated genes were considered (Stenton *et al.* 2024). Importantly, this analysis concluded that genome-wide calibration is unlikely to result in the inappropriate classification of an excessive number of variants as pathogenic or likely pathogenic. However, others have raised concerns about the limitations of genome-wide calibration, particularly in terms of obscuring performance across different genes and modes of inheritance (Dias *et al.* 2024, Isakov *et al.* 2024, Tejura *et al.* 2024). These findings suggest that genome-wide calibration alone may not be sufficient to address these discrepancies and highlight the need for a gene- and context-specific calibration method.

Multiplex assays of variant effects (MAVEs) provide an unbiased source of functional evidence (Weile and Roth 2018, Fowler *et al.* 2023, Rubin *et al.* 2025), offering huge potential to improve the classification of germline genomic variants, particularly rare missense variants frequently classified as uncertain significance (Chen *et al.* 2023). However, similar to the output of VEPs, raw scores from MAVEs vary in range and distribution across genes and do not readily correspond to ACMG/AMP evidence strengths.

Here, building on previous work (Loggerenberg *et al.* 2023, Gebbia *et al.* 2024, Isakov *et al.* 2024), we develop a method based on bootstrapped kernel density estimates to compute LRs for any functional score distribution—whether derived from MAVEs or VEPs—using pathogenic and benign truthset labels. The resulting LRs support robust and reproducible integration of functional scores from diverse sources into variant classification under ACMG/AMP guidelines. We have made our method available as an R package for high-throughput calibration studies, alongside a Google Colab notebook providing a simple interface for bench scientists.

## 2 Implementation

### 2.1 Likelihood ratio estimation

We follow van Loggerenberg *et al.* in using probability density estimation with a Gaussian smoothing kernel to derive LRs for functional scores (Loggerenberg *et al.* 2023), as the sliding window method developed by Pejaver *et al.* (2022) is not feasible at the gene level due to data sparsity. We set a lower limit on the number of variants in the truthset, requiring at least 10 benign and 10 pathogenic labels each. To avoid extrapolating LRs beyond the observed range, input scores are first clamped to the range of labelled scores, and then rescaled to the [0, 1] interval to ensure scale invariance. Density estimation is performed separately on the pathogenic and benign score distributions, using 1024 equidistant evaluation points and a bandwidth selected by biased cross-validation. This process is repeated 1000 times by resampling the reference distributions with replacement, applying kernel density estimation to each resample and projecting the resulting densities onto a common grid of 1024 points via linear interpolation. A fixed random seed is used to eliminate run-to-run variability.

Log-LRs are computed across resamples as the difference between pathogenic and benign log-densities at each index in the common grid, resulting in a 1000 × 1024 matrix. To stabilize estimates in sparse regions, we apply adaptive regularization:


$$\lambda_{i}=\frac{{\text{MAD}}_{i}}{{\text{max}}_{j}{\text{MAD}}_{j}}\cdot \frac{\sqrt{\sum_{j}{\left({\text{MAD}}_{j+1}-{\text{MAD}}_{j}\right)}^{2}}}{\sum_{j}{\text{MAD}}_{j}}$$

Here, MAD_*i*_ is the median absolute deviation of log-LRs at grid index *i*. The first term scales MAD values to reflect relative variability across the score range. The second term, a root-sum-of-squares of lagged differences, acts as a roughness penalty similar to L2 regularization. This formulation penalizes sharp or erratic fluctuations in the log-LRs while leaving stable regions largely unaffected.

**Figure 1.**
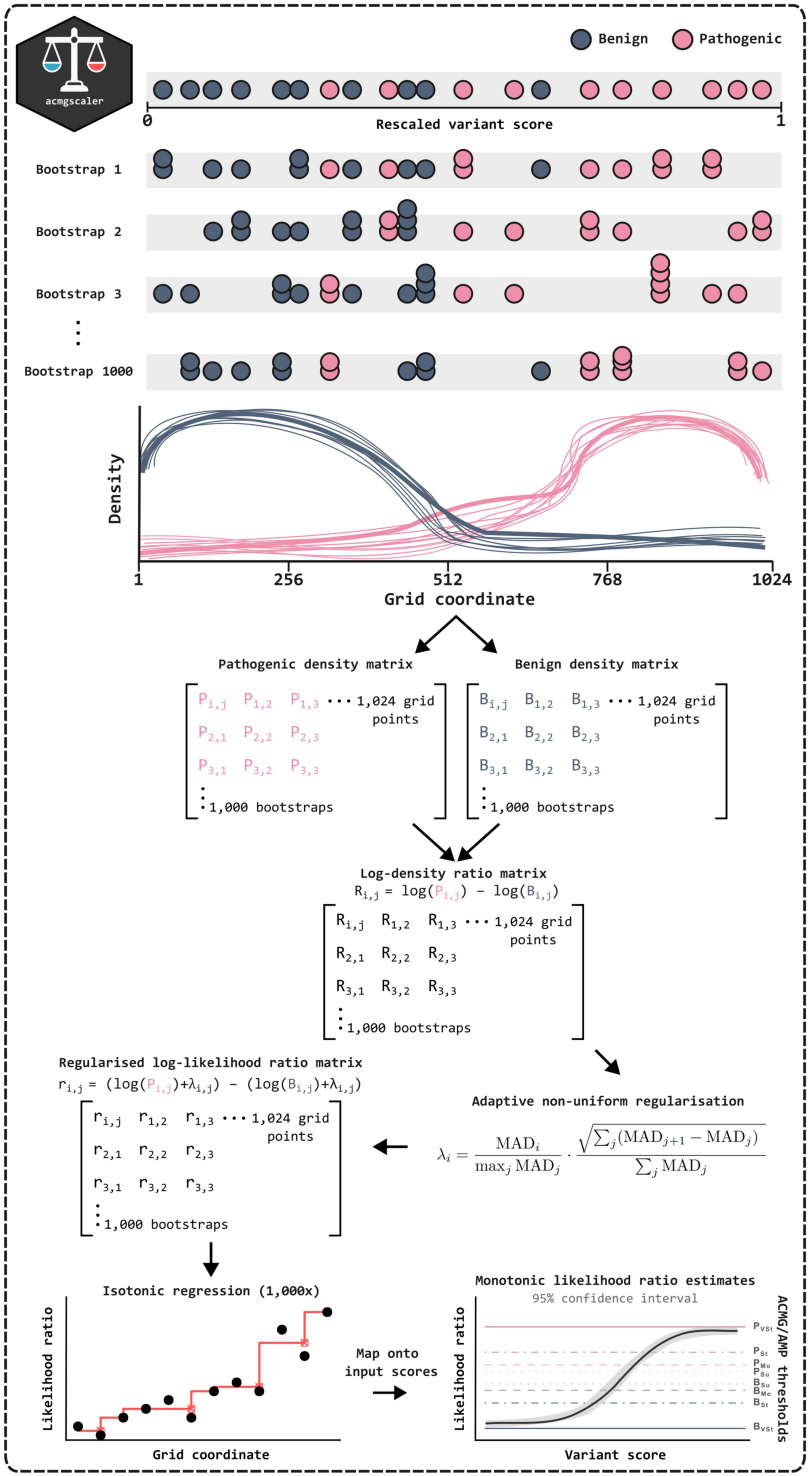
Method flowchart.

At this stage, the log-LRs may be non-monotonic due to boundary effects or multimodal structures in the score distributions, which could reflect noise, assay or VEP limitations, or inaccuracies in truthset labels. When higher scores indicate a greater likelihood of pathogenicity, a lower score should not yield a higher LR, as this would suggest local overfitting. To correct this, we apply isotonic regression to enforce monotonicity in the log-LR matrix, ensuring a gradual or stepwise increase in evidence across the score range. The direction of monotonicity is determined by the sign of the Spearman correlation between the column-wise mean of log-LRs and the input scores. The final LRs are obtained by computing the median log-LR across resamples, exponentiating to return to linear space, and mapping the estimates to the input scores based on their grid indices. A flowchart illustrating the method is shown in Fig. 1.

### 2.2 Computing confidence intervals for the likelihood ratios

We compute 95% confidence bounds for the population median of log-LRs across resamples by inverting the sign test, using the exact binomial distribution of order statistics (Conover 1999). An intuitive definition of this confidence interval is that if calibration were repeated 100 times with different random seeds, the median LR would fall within the interval in 95 of those repeats. The choice of a confidence interval for the location of the median log-LR, rather than, e.g. a percentile interval for the distribution of log-LRs, better aligns with our classification goal, as we care most about where the typical (central) log-LR lies. It is important to clarify that these confidence bounds do not represent uncertainty in the posterior probability of pathogenicity as defined in the ACMG/AMP framework. Instead, they reflect variability in the LR itself, estimated from the regularized and monotonic log-LR distribution. A classification based on a variant for which both the point estimate and the lower (for pathogenic) or upper (for benign) confidence bound exceed a given threshold is less likely to be affected by future truthset updates, e.g. the inclusion of additional pathogenic variants, than one based on the point estimate alone. Accordingly, we classify variants into evidence strengths using the lower bound when the point estimate exceeds one, and the upper bound when it falls below.

### 2.3 Minimum truthset size requirement

While as few as four samples can keep relative error low in one-dimensional kernel density estimation (Silverman 2018), we require a minimum truthset of 10 pathogenic and 10 benign variants to ensure broad generalizability across diverse distributions. We evaluated this choice using Monte Carlo cross-validation. Specifically, we selected genes (*N* = 187) with at least 20 missense variants classified as benign/likely benign (B/LB) and pathogenic/likely pathogenic (P/LP) in ClinVar (Landrum *et al.* 2020), requiring a minimum review status of one star. From each gene, we randomly sampled 10 variants per class without replacement and calibrated AlphaMissense (Cheng *et al.* 2023) and CPT-1 (Jagota *et al.* 2023) scores, which were selected based on their top performance in a recent benchmarking study (Livesey and Marsh 2025a). We repeated this process for 2000 trials. For each trial, we calculated the observed LR as the ratio of true pathogenic to true benign variants in a given ACMG/AMP evidence level, excluding those used for calibration. We then divided this value by the background ratio of true pathogenic to true benign variants in the gene.

**Figure 2.**
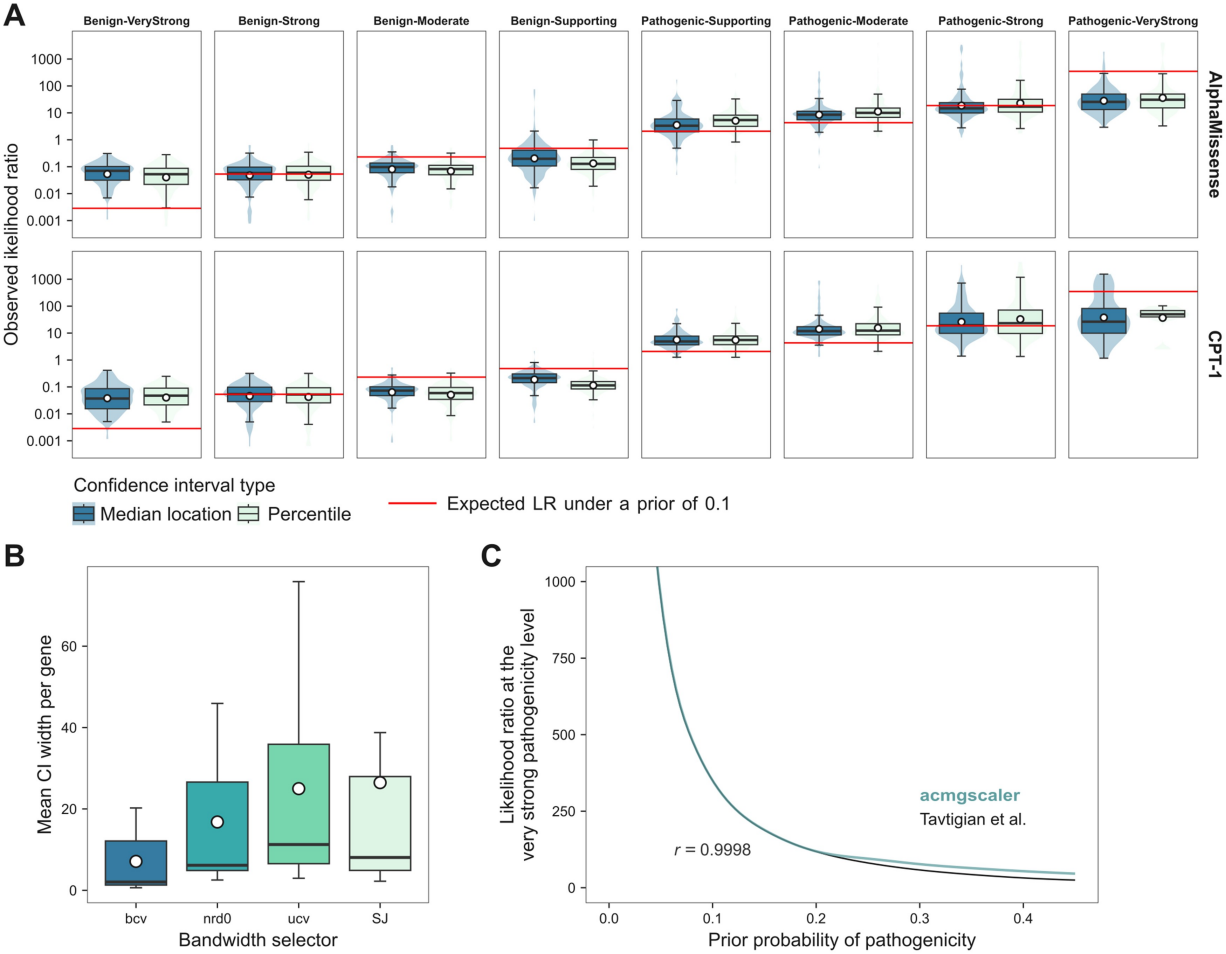
(A) Box-violin plots of the Monte Carlo cross-validation showing the distribution of observed LRs for 187 genes across ACMG/AMP evidence strengths and for AlphaMissense and CPT-1 scores separately. Each evidence strength category shows observed LRs with the classification either based on the median location or the percentile method for calculating confidence intervals. Boxes represent data within the 25th and 75th percentiles, with the middle line indicating the median, dots indicating the mean, and whiskers extending to 1.5× the interquartile range. (B) Comparison of bandwidth selection methods for computing LRs. The *y*-axis shows the gene-averaged width of the LR confidence interval across calibrated MAVE, AlphaMissense and CPT-1 scores. (C) Likelihood points for the very strong pathogenicity level as implemented in the acmgscaler package. The Pearson correlation (*r*) with the reference method by Tavtigian *et al.* is shown.

The aim of this analysis is to assess how well the calibrated thresholds recover expected evidence strengths when using the minimum sample size. For benign evidence, observed LRs should typically be at or below the expected value, and for pathogenic evidence, at or above it. This would show that the minimum sample size is not prone to inflating evidence strength due to chance variation in small truthsets. In Fig. 2A, we compare the observed LRs to the expected LR under a prior probability of 0.1. For the vast majority of genes, the observed LRs follow this pattern across the range from strong benign to strong pathogenic evidence. Caution is warranted for the very strong category, which is harder to assess due to data sparsity and occasional instability in the estimates. Nevertheless, these results demonstrate that the calibrated thresholds are generally reliable with the default minimum truthset size requirement.

Additionally, we used this simulation framework to compare the median location-based and percentile methods for computing confidence intervals. As shown in Fig. 2A, the resulting distributions differ only marginally, supporting the adoption of the median location-based method.

### 2.4 Choice of bandwidth selector

The bandwidth parameter controls the smoothness of the estimated density functions and can substantially affect the resulting LRs. We evaluated four automatic bandwidth selectors available in base R: ‘nrd0’ (Silverman’s rule of thumb), ‘bcv’ (biased cross-validation), ‘SJ’ (Sheather–Jones), and ‘ucv’ (unbiased cross-validation) (Sheather and Jones 1991, Sain *et al.* 1994, Silverman 2018). To select the most suitable method, we compared the average width of the LR confidence interval across truthset variants, which directly contribute to the density estimates, for seven human disease genes with available MAVE (Findlay *et al.* 2018, Zheng *et al.* 2022, Jia *et al.* 2021, Weile *et al.* 2021, Lo *et al.* 2023, Clausen *et al.* 2024, Kotler *et al.* 2018), AlphaMissense, and CPT-1 scores. All seven genes include at least 10 missense variants classified as B/LB and P/LP pathogenic in ClinVar, with a minimum review status of one star, which served as truthset labels for calibration. For each gene, the MAVE scoreset with the highest area under the receiver operating characteristics curve was selected. The genes, MAVE studies, scoreset sources, and the number of pathogenic and benign variants are listed in Table S1, available as supplementary data at *Bioinformatics* online. As shown in Fig. 2B, ‘bcv’ yielded the narrowest confidence intervals on average, suggesting greater stability, and was therefore set as the bandwidth selector in our method.

### 2.5 Determining likelihood points for a given prior

The prior probability of pathogenicity (hereafter ‘prior’) represents the estimated likelihood that a randomly selected, previously uncharacterized variant is disease-causing in a patient presented with a given phenotype before considering variant-specific evidence. Global estimates, such as 0.1 (Tavtigian *et al.* 2018) and the more recent 0.0441 (Pejaver *et al.* 2022), provide a useful baseline across diverse disease-associated genes, but priors may vary substantially between genes, and even among phenotypes. This variation reflects differences in gene properties, including their biological role, degree of functional constraint, associated disease prevalence, mode of inheritance, and molecular mechanism. To leave room for future advances in gene-specific prior estimation, the default prior of 0.1 can be replaced with a user-specified value.

Likelihood points corresponding to ACMG/AMP evidence strengths under a predefined prior can be determined using the framework introduced by Tavtigian *et al.* (2018). Here, we approximate this by considering that the *P_ii_* and *LP_i_* combining rules yield internally inconsistent likelihood points: *P_ii_* produces a posterior probability below the expected 0.99 threshold, whereas *LP_i_* exceeds it. To determine the very strong pathogenicity threshold, we identify the smallest LR that satisfies at least 13 of 14 posterior probability criteria across the *LP* and *P* combining rules. Although the original ACMG/AMP framework imposes additional conditions—requiring the *LB* combining rules to have a posterior probability between 0.05 and 0.001, and the *B* combining rule to be below 0.001—we find that enforcing the criteria for *P* and *LP* implicitly maintains a similar relationship between the prior and the LR threshold for the very strong pathogenicity evidence (P_*VSt*_). As shown in Fig. 2C, for priors below 0.2, our method closely aligns with the proposed combining rules, yielding slightly more conservative values above 0.2. Notably, for the prior of 0.1 used by Tavtigian *et al.* which gives a P_*VSt*_ threshold of 350, our approach yields the same value. For the prior of 0.0441 suggested by Pejaver *et al.* which corresponds to a P_*VSt*_ threshold of 1124, our estimate is 1131. A difference of seven LR points is negligible in practice, as it represents only 0.64% of the range between the strong and very strong evidence thresholds under this prior.

### 2.6 Deriving score thresholds for evidence levels

To compute score intervals for the evidence levels, we first determine P_*VSt*_ under the specified prior and derive the corresponding likelihood points for the remaining levels. For each evidence strength, the score threshold is obtained by linearly interpolating the score at which the lower confidence bound of the LR crosses the evidence threshold if the point estimate exceeds one, and the upper bound otherwise. Importantly, the interpolation is done on the common grid coordinates, ensuring that the values are derived from the full resolution of the LR curve.

### 2.7 Calibration examples

**Figure 3.**
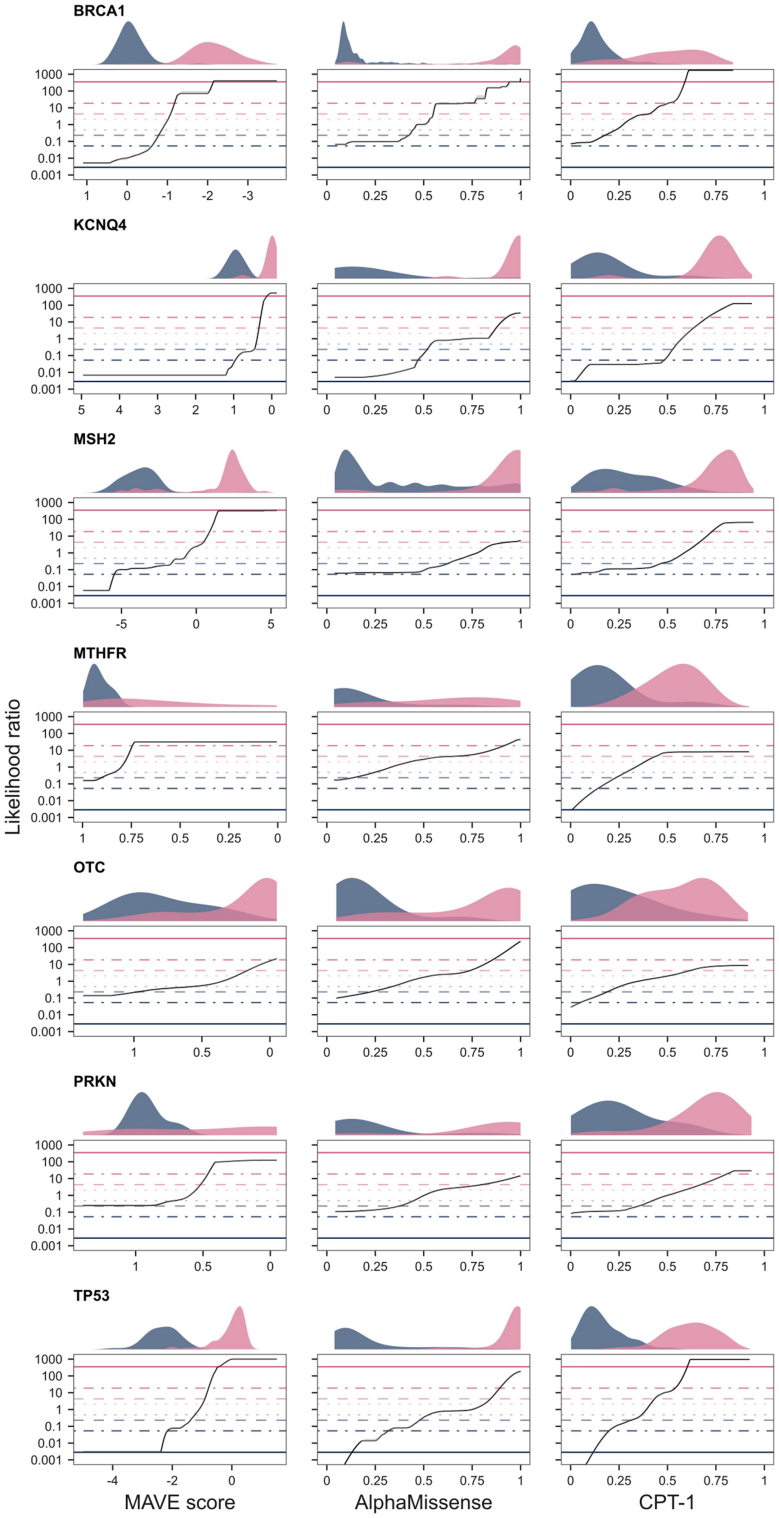
Calibrated MAVE assay, AlphaMissense and CPT-1 scores for missense variants in human genes using acmgscaler. Density plots show the distribution of pathogenic (pink) and benign (blue) scores. In MAVE assays, the *x*-axis is inverted for BRCA1, KCNQ4, MTHFR, OTC, and PRKN for consistent directionality. Where visible, shaded areas represent 95% confidence intervals. Horizontal bars show evidence strengths under a prior of 0.1, ranging from pathogenic very strong at the top to benign very strong at the bottom. Note that the *x*-axis is log-scaled with raw LR tick labels, such that log-LR = 0 corresponds to LR = 1 (where the odds of pathogenicity equal those of benignity, defining the log-midpoint of the indeterminate evidence level).

Full calibration profiles for the seven genes, using a global prior of 0.1, are shown separately for MAVE assays, AlphaMissense, and CPT-1 scores in Fig. 3. Despite substantial differences in distribution shape, modality, and scale across score sets, the LR estimates generally track well with the empirical density distributions of pathogenic and benign variants. This alignment suggests that the method is robust to underlying functional score characteristics and captures consistent evidence strengths across both experimental and computational predictors. While the confidence intervals may appear narrow, this reflects the robustness of the central tendency of the LR estimates. As a complementary visualization, we show percentile-based confidence intervals in Fig. 1, available as supplementary data at *Bioinformatics* online, which better reflect the overall variance of the resampled log-LR distribution but are less aligned with our classification objective, which depends on the location and uncertainty of the central estimate.

To showcase context-specific calibration, we examined the MTHFR assay conducted in a p.Ala222Val background at different folinate concentrations (Weile *et al.* 2021). The p.Ala222Val variant is associated with reduced enzyme activity and thermostability (Frosst *et al.* 1995), yet it is also common, with carrier frequencies approaching 50% in some ancestry groups. Individuals homozygous for the allele display elevated homocysteine levels (hyperhomocysteinaemia), a hallmark of folate-cycle disruption, which can be ameliorated by folate supplementation (Marini *et al.* 2008). The p.Ala222Val background captures both genetic and nutritional context-dependent effects, which are essential for accurate functional annotation and clinical interpretation of other MTHFR missense variants. To calibrate these assays, we used early-onset hyperhomocysteinaemia variants collected by the authors, and appended ClinVar benign variants with population variants observed at least once in the homozygous state in gnomAD (Chen *et al.* 2024). This truthset makes it possible to run the calibration separately on the catalytic and regulatory domains, with both meeting the minimum truthset size requirement.

**Figure 4.**
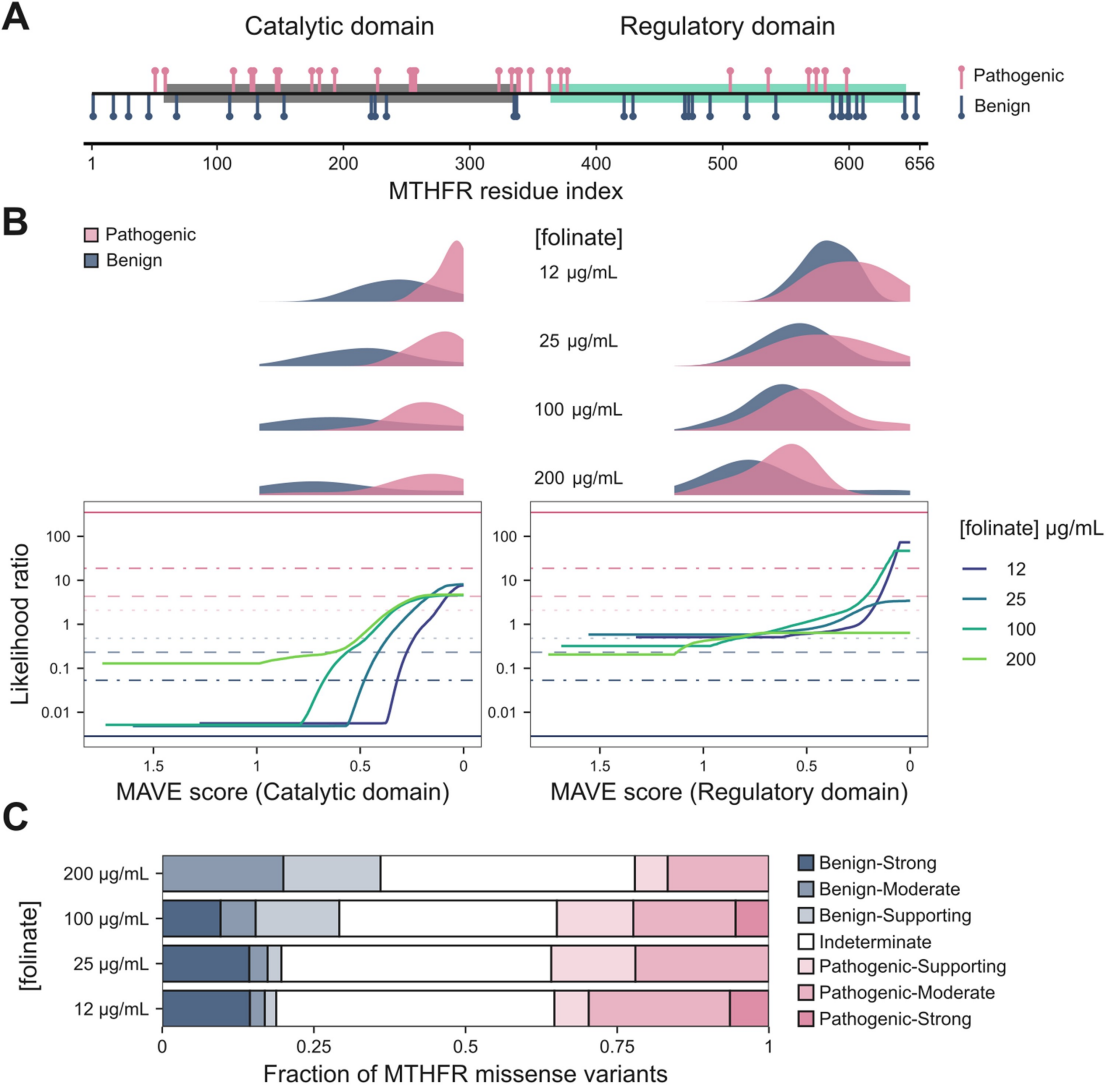
Calibration results for MTHFR MAVE scores in the p.Ala222Val background conducted at different folinate concentrations by Weile *et al.* (2021). (A) Location of pathogenic and benign truthset variants along the MTHFR sequence, with the catalytic and regulatory domains highlighted. (B) MAVE score versus LR plots at different folinate concentrations. The density distributions of truthset variants are shown on the top. (C) Composition of ACMG/AMP evidence classes for all possible MTHFR missense variants with a MAVE score, subset by folinate concentration.

Figure 4A and B shows the result of the calibration. Consistent with previous findings, scores in the regulatory domain tend to be predictive only of pathogenicity, whereas scores in the catalytic domain can support moderate evidence of pathogenicity and strong evidence of benignity. While the original study reported stronger pathogenic evidence from catalytic domain scores, this discrepancy may reflect differences in the truthset or the effect of our more conservative regularization, which penalizes high-variance estimates more strongly. Despite this, we observe a clear folinate concentration-dependent effect on ACMG/AMP evidence strengths: lower concentrations yield a higher fraction of missense variants classified as pathogenic and a lower fraction classified as benign, both with a shift towards stronger levels of evidence (Fig. 4C). Thus, our method successfully recapitulates gene-specific effects observed in the MTHFR study, and illustrates how incorporating additional context into calibration frameworks can improve variant interpretation.

### 2.8 Genome-wide versus gene-specific thresholds

**Figure 5.**
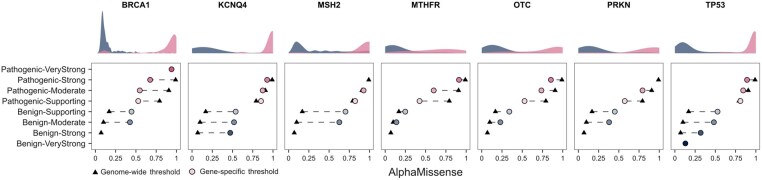
Genome-wide versus gene-specific AlphaMissense thresholds for the seven genes shown in Fig. 3. Density plots show the distribution of pathogenic (pink) and benign (blue) scores. Dashed lines represent a shift between genome-wide and gene-specific thresholds.

In Fig. 5, we compare genome-wide AlphaMissense thresholds (Bergquist *et al.* 2025) with gene-specific thresholds derived for the seven genes shown in Fig. 3. Notably, gene-specific calibration reveals that MSH2 and PRKN do not attain strong pathogenicity evidence, and that BRCA1, MSH2, MTHFR, OTC, and PRKN likewise fail to reach strong benign evidence. In contrast, BRCA1 reaches very strong pathogenic evidence and TP53 reaches very strong benign evidence when thresholds are determined at the gene level. Overall, genome-wide and gene-specific thresholds vary substantially: in every case where both exist, the genome-wide benign threshold is lower. Moreover, in KCNQ4, MSH2, and TP53, the supporting pathogenicity thresholds are slightly higher than their genome-wide equivalents. This pattern suggests that, while genome-wide thresholds are unlikely to overcall pathogenic variants in these genes, adopting gene-specific thresholds would enable a greater proportion of variants to be classified as benign. These results further stress the importance of considering the unique functional landscapes of genes when applying calibration thresholds.

## 3 Colab notebook

**Figure 6.**
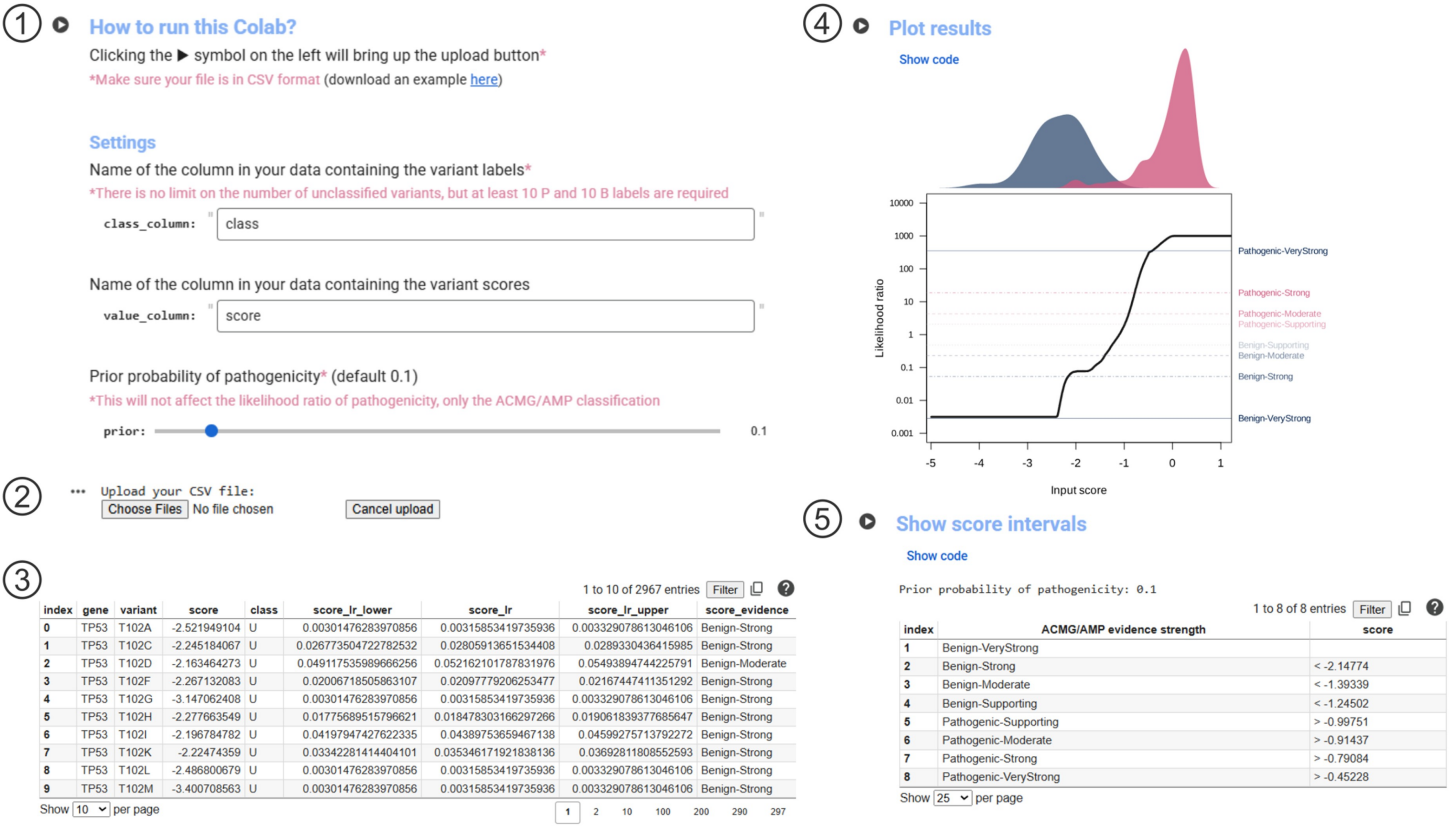
Colab notebook for functional score calibration and variant classification. (1) Input fields. (2) Upload button. (3) Interactive results table. (4) Visualization of the input score versus LR curve. (5) Display of score thresholds for evidence levels.

We have created a Google Colab interface for acmgscaler, allowing calibration of custom datasets without the need to install R. The input form, along with an example calibration output, is shown in Fig. 6. The available settings are simplified to a numeric score column and a class column containing the truthset labels. The class column should include at least 10 instances of ‘P’ (pathogenic/positive class) and ‘B’ (benign/negative class) labels each. For any other or missing labels, LRs will also be computed. A custom prior can be set, influencing the evidence strength-based classification results. Upon running the cell, an upload button appears to provide data in CSV format. Once calibration is complete, the Colab automatically downloads the results, while the interactive table allows data export in different formats or filtering through a simple graphical interface. A separate cell enables visualization and export of the input score versus LR curve, with the corresponding ACMG/AMP evidence thresholds highlighted under the predefined prior. The score thresholds can be displayed and exported for downstream use.

## 4 R package

### 4.1 Installation

The development version of acmgscaler can be installed using devtools:

# install devtools if not installed yet

**install.packages** ( 'devtools' )

# install the package from the GitHub repository

**devtools::install_github** ( 'badonyi/acmgscaler' )

### 4.2 Usage

The main function calibrate() adds LRs as new columns to an existing dataframe containing the variant scores and the truthset labels, and derives the score thresholds for the ACMG/AMP evidence strengths. For convenience, these are computed for all numeric columns if value is set to NULL. The function returns a named list with two elements:

- likelihood_ratios: the input dataframe appended with LR estimates and their confidence bounds (<value>_lr_lower, <value>_lr, <value>_lr_upper), and a column with assigned ACMG/AMP classifications under the set prior (<value>_evidence).
- score_thresholds: the lower and upper score bounds defining each evidence strength.

If a grouping variable is supplied, calibrate() returns a nested list of the same structure for each group. The example data included in the package contains MAVE scores for BRCA1 and TP53 variants and their class labels from this study, and can be calibrated as follows:

**library**(acmgscaler)

# load example data provided with the package

**load**(variant_data,package ='acmgscaler')

variant_data_calibrated <-  **calibrate**(

  df =variant_data,

  # column containing the variant scores (optional)

  value ='score',

  # grouping variable (optional)

  group ='gene',

  # prior for classification (default 0.1)

  prior = 0.1

)

# LRs for BRCA1 variants

**head**(variant_data_calibrated$BRCA1$likelihood_ratios)

# evidence thresholds for BRCA1 scores

**head**(variant_data_calibrated$BRCA1$score_thresholds)

## 5 Discussion

We developed a functional score calibration method within the ACMG/AMP framework. This approach is entirely data-driven, eliminating arbitrary thresholds and manual tuning, and yields stable LRs across diverse assay types and computational predictors, regardless of distribution shape or scale.

Our methodology builds on the core idea behind maveLLR, a kernel density estimation method developed to assign ACMG/AMP evidence strengths for MAVE assays (Loggerenberg *et al.* 2023, Gebbia *et al.* 2024). We extend this framework with resampling, which permits the use of a variance-based penalty to shrink unstable estimates towards one and to compute confidence bounds. Monotonicity constraints on the LRs are enforced via isotonic regression, introducing only modest, local adjustments to the curve and preserving its empirically supported structure. The combination of adaptive shrinkage and a simplified thresholding procedure ensures that the assigned evidence strengths remain robust and conservative. These steps are implemented entirely in base R using highly optimized core functions, maintaining long-term backwards and forwards compatibility. Moreover, we provide a Google Colab wrapper, bypassing the need to install R for single custom datasets.

Zeiberg *et al.* recently introduced an alternative calibration framework for MAVE assays based on a skew-normal mixture model (Zeiberg *et al.* 2025). This method fits score distributions using parametric components, with monotonicity enforced via an expectation–maximization algorithm. By jointly estimating parameters across the entire score distribution, this approach is particularly effective at stabilizing sparse or extreme score regions. The authors also propose a strategy for estimating the prior (the probability that a variant in a patient with a given phenotype is causal for the disease, before incorporating evidence) by modelling gnomAD (Chen *et al.* 2024) scores as a mixture of pathogenic and benign components. In contrast, our framework computes LRs directly from empirical distributions of labelled variants, without relying on parametric assumptions, making it readily generalizable to any continuous score set, including those of VEPs. A standardized calibration pipeline for both MAVE and VEP data ensures all functional scores are treated identically, making evidence integration and auditing straightforward within the ACMG/AMP framework.

The sole parameter in our method is the prior. While genome-wide priors have been estimated before (Tavtigian *et al.* 2018, Pejaver *et al.* 2022), deriving reliable context-specific priors remains challenging. This is because Mendelian disease genes frequently have multiple phenotypes, which may differ in their population prevalence, mutational spectrum (Chong *et al.* 2015) and molecular mechanism (Badonyi and Marsh 2025). Even in genes associated with a single phenotype, estimating priors from population data, such as gnomAD, can be problematic. For example, genes with recessive conditions typically have a limited number of alleles that have been observed in the homozygous state, which represent a more suitable proxy for benign variants in these genes than heterozygous alleles (Gerasimavicius *et al.* 2022). We anticipate that with increasing population sequencing data and advances in statistical genetics methods, reliable estimation of context-specific priors suitable for practical application will be feasible in the near future. Until then, we recommend using a constant prior, e.g. 0.1, to avoid the risk of inflating evidence strengths as a result of overestimating the prior.

We developed our method with missense variants in mind, as most VEPs are limited to them. However, the approach can in principle be applied to any variant type, provided the scores are derived from the same assay or VEP and reflect quantitatively comparable functional consequences. Particular care is needed when including nonsense variants from MAVEs. For the majority of MAVE approaches that rely on exogeneous constructs, a premature stop codon will result in a truncated protein product, which may not mimic the biological effects within cells where nonsense-mediated decay is often involved. Similarly, certain MAVEs may not detect aberrant splicing events (Livesey and Marsh 2025b); thus, users should evaluate whether the assay setup captures the relevant biology for each variant type when performing calibration.

Another important consideration is the potential for circularity in the calibration dataset. We used ClinVar-labelled pathogenic and benign variants as the truthset to calibrate MAVE and VEP scores across seven human genes. However, many of the MAVE assays may have already contributed functional evidence towards these clinical classifications (Fortuno *et al.* 2021, Parsons *et al.* 2024), raising the possibility of inflating the calibrated evidence strength. VEPs are similarly susceptible to this form of bias (Grimm *et al.* 2015). For example, AlphaMissense was trained in part on population allele frequencies, which inform benignity assessments under ACMG/AMP guidelines (Harrison *et al.* 2019), although the resulting bias appears modest (Pathak *et al.* 2024, Livesey and Marsh 2025a). CPT-1 was partially trained on MAVE data, including the assay for the MTHFR gene, yet showed no strong evidence of bias in our calibration. To mitigate circularity, truthsets ideally should exclude variants whose classification relied on the predictive features being evaluated. As MAVEs and VEPs are increasingly incorporated into clinical variant interpretation, such circularity is likely to become more pervasive. It will therefore be essential to track the provenance of supporting evidence to ensure robust calibration and reliable downstream use. Sources of potential circularity warrant careful consideration in calibration projects intended to inform clinical decision-making.

For simplicity, we used ClinVar-labelled pathogenic and benign variants without stratifying by disease phenotype or inheritance mode. However, such context-specific considerations are critical for accurate clinical calibration and are already adopted by variant curation expert panels. Researchers with gene-specific knowledge often have access to additional pathogenic labels from alternative databases, detailed literature reports, patient pedigrees, and case-control studies. While author-curated reference sets may suffice for exploratory analyses, calibration to clinical genetics standards requires high-quality disease variants defined through expert consensus. Nonetheless, the availability of bona fide benign labels is often more limiting. While allele frequency cutoff-based approaches may yield useful proxy-benign variants for highly constrained dominant genes (Xu *et al.* 2023, Axakova *et al.* 2025, Rowlands *et al.* 2025), caution is required for recessive conditions where heterozygous population variants can drive disease. Although VEP-based thresholds could offer an alternative, in supervised models they depend on the training data, which can thus influence the resulting calibration in ways that overstate confidence for variants similar to the training set while underestimating risk for novel or underrepresented variants. Ultimately, large-scale population sequencing remains the most viable avenue to augment benign reference sets and accelerate the discovery of disease-associated variants.

## Supplementary Material

btaf503_Supplementary_Data

## Data Availability

The calibration data underlying this article are available in the OSF repository at https://osf.io/7hjnm/.
