# Supplementary material for: acmgscaler: an R package and Colab for standardized gene-level variant effect score calibration within the ACMG/AMP framework
Source: Bioinformatics. 2025 Sep 10;41(10):btaf503. doi: 10.1093/bioinformatics/btaf503 (PMC12496131; doi:10.1093/bioinformatics/btaf503)
Supplement: btaf503_Supplementary_Data [file btaf503_supplementary_data.zip › tables1.docx]

**Table S1.** Sources of MAVE datasets calibrated in **Figure 3**. Either the MaveDB ID or the score name from the original publication is given. *N*_b_ and *N*_p_ are the number of benign and pathogenic missense mutations in the datasets, respectively. Note that AlphaMissense and CPT-1 have full coverage in these genes with equal or greater labelled variant counts.

| **Gene** | **Reference** | **Score name** | **MaveDB ID** | ***N*_b_** | ***N*_p_** |
| --- | --- | --- | --- | --- | --- |
| BRCA1 | Findlay *et al.* 2018 | function.score.mean |  | 82 | 158 |
| KCNQ4 | Zheng *et al.* 2022 |  | 00000094-a-2 | 10 | 13 |
| MSH2 | Jia *et al.* 2021 |  | 00000050-a-1 | 242 | 127 |
| MTHFR | Weile *et al.* 2021 |  | 00000049-a-1 | 11 | 32 |
| OTC | Lo et al. 2023 |  | 00000112-a-1 | 10 | 94 |
| PRKN | Clausen *et al.* 2024 |  | 00000114-a-1 | 16 | 15 |
| TP53 | Kotler *et al.* 2018 | RFS_H1299 |  | 37 | 182 |
