# Supplementary figures and images for: acmgscaler: an R package and Colab for standardized gene-level variant effect score calibration within the ACMG/AMP framework
Source: Bioinformatics. 2025 Sep 10;41(10):btaf503. doi: 10.1093/bioinformatics/btaf503 (PMC12496131; doi:10.1093/bioinformatics/btaf503)

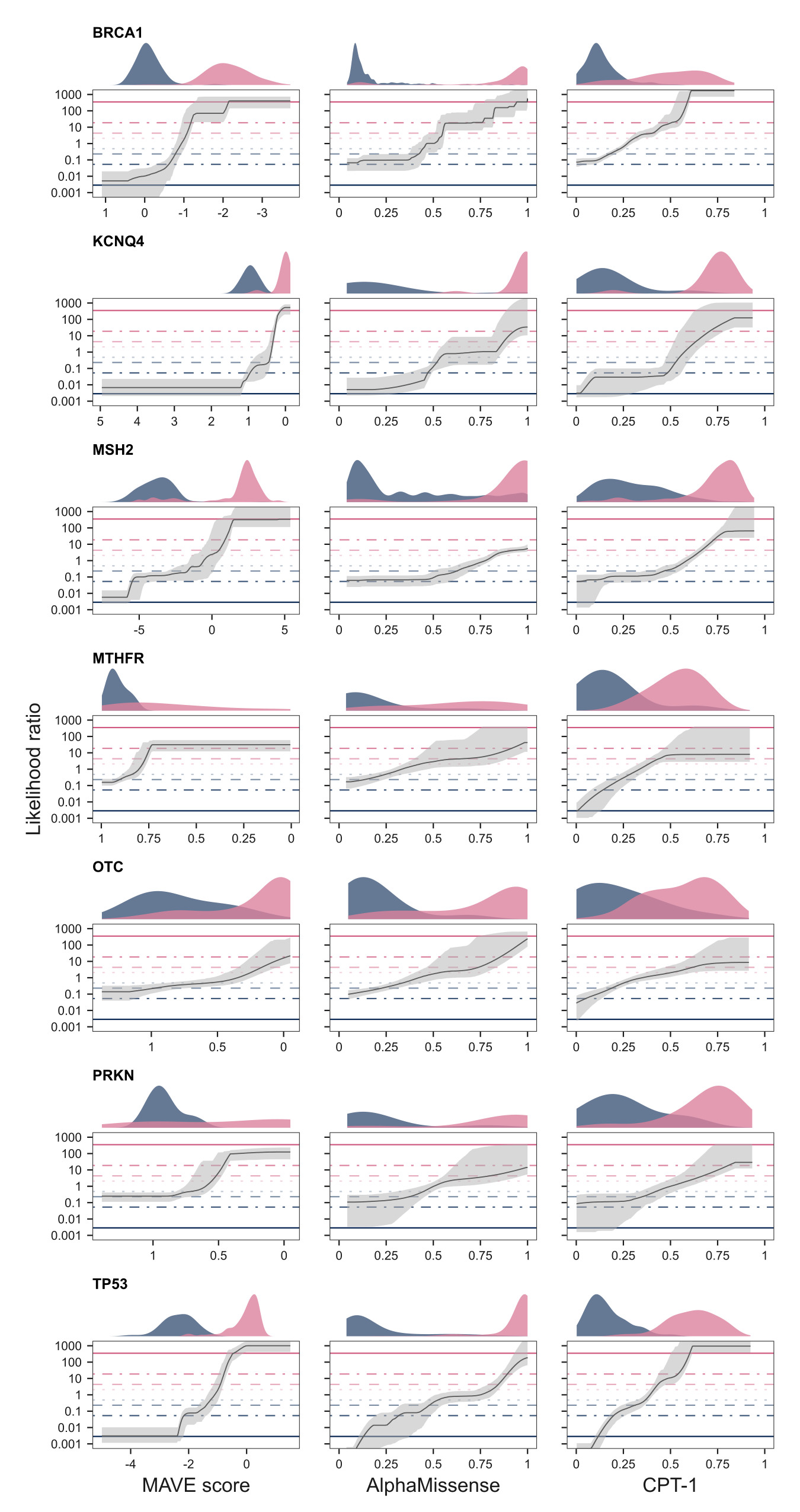

Supplement: btaf503_Supplementary_Data [file btaf503_supplementary_data.zip › figs1.tiff]
